# Organ-on-a-chip: current gaps and future directions

**DOI:** 10.1042/BST20200661

**Published:** 2022-04-19

**Authors:** Pelin L. Candarlioglu, Gianni Dal Negro, David Hughes, Frances Balkwill, Kate Harris, Hazel Screen, Hywel Morgan, Rhiannon David, Sonja Beken, Olivier Guenat, Wendy Rowan, Augustin Amour

**Affiliations:** 1 In Vitro/In Vivo Translation-Complex In Vitro Models, GlaxoSmithKline, Gunnels Wood Road, Stevenage, Hertfordshire SG1 2NY, U.K.; 2 CN Bio Innovations Ltd, Cambridge, U.K.; 3 Barts Cancer Institute, Queen Mary University of London, London, U.K.; 4 National Centre for the Replacement, Refinement and Reduction of Animals in Research (NC3Rs), London, U.K.; 5 School of Engineering and Material Science, Queen Mary University of London, London, U.K.; 6 Electronics and Computer Science, University of Southampton, Southampton, U.K.; 7 Safety Innovation, Clinical Pharmacology & Safety Sciences, R&D, AstraZeneca, Cambridge, U.K.; 8 Federal Agency for Medicines and Health Products, Brussels, Belgium; 9 ARTORG Center, University of Bern, Bern, Switzerland; 10 Novel Human Genetics Research Unit GlaxoSmithKline, Gunnels Wood Road, Stevenage, Hertfordshire SG1 2NY, U.K.; 11 Immunology Research Unit, GlaxoSmithKline, Gunnels Wood Road, Stevenage, Hertfordshire SG1 2NY, U.K.

**Keywords:** drug discovery and design, organ on chip, translational science

## Abstract

As an emerging hot topic of the last decade, Organ on Chip (OoC) is a new technology that is attracting interest from both basic and translational scientists. The Biochemical Society, with its mission of supporting the advancement of science, with addressing grand challenges that have societal impact, has included OoC into their agenda to review the current state of the art, bottlenecks and future directions. This conference brought together representatives of the main stakeholders in the OoC field including academics, end-users, regulators and technology developers to discuss and identify requirements for this new technology to deliver *on par* with the expectations and the key challenges and gaps that still need to be addressed to achieve robust human-relevant tools, able to positively impact decision making in the pharmaceutical industry and reduce overreliance on poorly predictive animal models.

## Introduction

The high attrition rate the pharmaceutical industry is still facing indicates that the current tools for conducting research too often fail in predicting the outcome in patients [1]. Although considerable advances have been made in understanding the pathophysiological differences and similarities between patients and pre-clinical animal models, there are still instances of failure in predicting clinical results in particular when addressing efficacy and toxicity [2]. To close these gaps, the success rate of clinical trials for new medicines and vaccines needs to be increased by improving predictive capabilities for more accurate decisions starting from the early stages of drug development. This transformative drug development landscape also requires the identification of new biomarkers to diagnose diseases and to assess treatments, as well as new human-relevant tools to assess the efficacy and safety of future medicines. The existing gaps in translating preclinical findings to the clinic have been the main drivers for biologists and engineers in leveraging recent advances in tissue engineering, stem cell research and micro-fabrication to develop novel Complex In Vitro Models (CIVMs) that more closely mimic pathophysiological functions of human tissues and organs. Organ on chip (OoC) is a type of CIVM that is defined as ‘a fit-for-purpose microfluidic device, containing living engineered organ substructures in a controlled microenvironment, that recapitulates one or more aspects of the organ's dynamics, functionality and (patho)physiological response *in vivo* under real-time monitoring' by the Organ-on-Chip In Development (ORCHID) initiative, EU funded consortium as part of the Horizon2020 project [3].

OoCs are generally developed by investor-backed and moderately sized start-ups with less than five years of existence [4]. There is also some technology development within academic institutions that is generally specialised in human tissue engineering. They are focused on technology development at the prototype stage and publication of proof of concept data, with commercialisation being a secondary objective, typically achieved through spin-out companies or licencing. Besides, the qualification of a new technology needs to be tailored on the specific needs of the end-users; this aspect makes the dialogue and collaboration between technology developers and end-users critical for ensuring the products address needs and expectations.

The validation of a new *in vitro* model has been historically based on the assessment of three parameters: reliability, reproducibility and predictivity. Although these parameters are still critical, in the last decade, a dramatic paradigm shift has occurred in the way a new model is evaluated, since both regulators and scientists have reached the consensus that no model can be expected to universally address every possible biological question. In other words, science and technology is not yet advanced enough to artificially reproduce all the complexity of a living organism. This assumption has led to a new, more focussed way of evaluating a model for a specific area or application by restricting the assessment of its predictive capacity to a limited context of use. In the context of medicinal products, the way of demonstrating scientific validity is referred to as *qualification* and is nowadays broadly embraced by both the scientific community and regulators. Qualification aims to convince both regulators and end-users that the CIVMs are suitable or ‘fit for purpose' for answering a particular question in a defined context of use (CoU), with clear qualification criteria.

This report describes the main findings and conclusions of the ‘Organ-on-a-chip: Current Gaps and Future Directions' conference organised by the Biochemical Society together with GSK, during which new ideas for OoC based non-animal approaches in the assessment of safety and efficacy of new therapies were discussed. The conference was held on the 2nd of December 2019 in Stevenage, U.K. This conference aimed to layout the current challenges in adopting these models, formulating the strategic elements to overcome these challenges by deriving a list of recommended actions and setting out realistic expectations about the use of these models in near future (Table 1).

**Table 1:** Key messages from the conference portraying the current state of the field and the future roadmap

Key opportunities, challenges and solutions to improve the field: lessons from the panel discussion				
Bottlenecks	Bigger picture	Future opportunities	Key questions for future	Proposed actions To further develop alternatives
• Very crowded field, hard to distinguish early on what will have longevity • Current model development timelines are too long, more momentum is needed. • Creating separate models for each case is not sustainable. Need to combine safety and efficacy models • Communication and interaction between networks are fragmented • Data sharing between end-users and other stakeholders is still poor • Endpoint specific performance criteria with open source positive and negative reference compounds are lacking for most context of use, hindering unified qualification • Access to the right human tissue that is of good quality, functional and representative of the physiology is a challenge • Technology readiness level of most OoC models is not ready for wider adoption • Support for OoC developers (academic and/or start-ups) from end-users or regulators is limited	• Examples of pharma integration into workflow are appearing which were not there a few years ago • Regulators are open to see combined safety and efficacy on the same model • Using OoC might be costly but compared with the cost of failed animal experiments or failed clinical trials, that is negligible. If translatable, the value is self-evident and worth the investment. • Complexity in system shouldn't be the complexity in use, it should be simple to use and robust. • Need to engage with the regulators early on. Multidisciplinary nature of OoC technology has more potential to capture human relevance and that is a factor for increased confidence of regulators • Applicability of clinical biomarkers for qualification endpoints will increase confidence because they are established validated biomarkers that are already in use. This will increase the translatability and the performance evaluation of these models.	• Possible to see OoC generated data as part of IND dossier in near future • Fully validated model with patient samples can be used for *in vitro* clinical trials • Need to increase the throughput to be able to do clinical trials on chip • Combining OoC with other methods since OoC is part of a spectrum and cannot answer every question. The real value comes from enhanced predictability together with *in silico* and *in vivo* data. • Precision medicine will be the field where these models will gain traction in the next 5–10 years. This is the value creation proposition for the field — to be able to look at patient samples or a subsection of patient population to define/refine/invent therapy • Developing live monitoring technology to get more data from one model real-time with multiple readouts	• Defining Context of Use is crucial to establish *fit for purpose* and subsequent qualification of the model. Main areas of interest here are *disease mechanism, efficacy, toxicity* and *personalised medicine*. • Replacing animals are feasible with OoC where one parameter is tested in a controlled manner preserving complexity whilst combining with simplicity. • Training regulators is important and an ongoing process but keeping the training consistent with same materials etc. matters. • Qualification criteria needs to be flexible since OoC is a dynamic field with constant progress. Key requirements are: - Well defined protocol - Defined context of use and the clear relevance showing the accuracy and also the limits of the method - Show reliability and robustness	• Regulators are the judge and you are trying to answer their questions so allow them to tailor your development accordingly. They also have oversight on every drug modality and can help with giving insight into the tools that are needed or to identify the gaps. • Data submission to regulators is encouraged • Improve and centralise data sharing to achieve progress • Establishing a set of commercially available set of reference compounds to create comparable validation data and assess the limitations of the model • Different networks and stakeholders need to communicate with each other, to prevent replication and enable harmonisation of protocols and get qualification together • More funding opportunities to specifically support qualification/validation studies

The Organ-on-a-chip: Current Gaps and Future Directions conference was designed to capture a realistic snapshot of the field as it stands and brought together participants from industry and technology developers, along with invited representatives from regulatory agencies, funding bodies and researchers from academia. The invited participants represented the Federal Agency for Medicines and Health Products (FAMHP, Belgium), the National Centre for the Replacement, Refinement and Reduction of Animals in Research (NC3Rs, U.K.), as well as pharmaceutical companies and OoC developers.

This report summarises the key points and take-home messages based on presentations and panel discussions with delegates at the conference, with the main focus on the practical issues of developing human relevant and clinically translatable *in vitro* models to increase the prediction of preclinical and clinical research and reduce reliance on animal models. This report should not be considered a complete or comprehensive review of research efforts in the area of OoC model development or alternatives to animal models, nor a detailed record of all discussions held, but rather a reflection on the strategic ideas that emerged to push the science forward and increase the pace of uptake.

## Organ on chip: when to use defines how to use

There is potential for OoC to be applied across the whole drug discovery and development workflow, however it is important to establish what models are suitable for which stage, as there are different requirements depending on whether the model will be applied in early drug discovery or at a later stage of drug development, such as before or in parallel to the clinical trials. While this might look like a trivial distinction, it indeed necessitates the CoU to be established, and drives the requirements with respect to qualification of the models. Moreover, when these models will be used defines whether any regulatory body interaction is required or not. If the model is used in preclinical drug development and there is an intention of using these data as part of a regulatory submission, guidance from regulatory bodies is critical to ensure acceptance of these data. However, if the model is used in the early drug discovery phase and no regulatory interaction is expected, then definitions can become more varied and specific to individual companies. This situation sums up the division in the field and represents some of the challenges that were discussed at the conference.

## OoC in early stage research and drug discovery

OoC models may have various levels of complexity and different throughputs. Due to this wide range, they find multiple plug-in points where they can be used in early drug discovery. Highly complex but lower throughput models enable human-specific pathway analysis for target identification or a thorough investigation of the mechanism of action at the target validation phase followed with high throughput screening to identify desirable compounds. After this stage, any initial hits identified can be validated further with OoC models that are higher throughput but still more complex than 2D systems to identify lead molecules. Later, at the lead optimisation and candidate selection phase, these OoC models support decision making for more effective and safer candidates to be progressed further into the development phase [5]. For target validation, candidate selection and safety assessment, the low throughput of the complex systems is not necessarily a bottleneck *per se* as recapitulating the multiparametric readouts of the human *in vivo* healthy and disease situation is more important which are reserved to animal models. Nevertheless, in drug lead discovery it would be beneficial to have systems with increased throughput retaining all the complexity necessary for replicating the *in vivo* conditions whilst enabling parametric assessments. Due to their complexity and similarly to animal models, OoC systems may present slightly higher variability than 2D models in same replica. However, unlike for animal models, there are no ethical concerns of increasing the n number and statistical power of an OoC experiment. In that regard, increasing OoC throughput whilst reducing experimental variability and running costs will enable to deploy these models more widely than is the case for animal models.

It should be considered, however, that in the early drug discovery phase, data are mostly kept internally to the individual company and are rarely shared externally, including with regulatory bodies. Currently, decisions on what constitutes the *fit for purpose, context of use* (see Box 1) and *validation/qualification* criteria is an internal issue for each individual company, which often has an internal process to address these questions, in much the same way an *in vivo* disease model would be evaluated. Qualification of these models is usually a small-scale exercise conducted in-house by the end-users (e.g. pharma) in collaboration with the model developers. This individualistic behaviour of end-users is a hurdle towards reaching consistency and harmonisation amongst the multiple end-users about qualification methodology. A clear need here is finding routes to publish and share these model qualification methods to standardise the field for all stakeholders.

**Definitions of key terminology**

**Context of use** is a clear definition of the scientific question where this method will be applied, how this test method will answer the question asked.

It needs to define the endpoints that will be measured in relation to the conventional endpoints, either human or animal, what measurement types these would be (biomarkers, histology, morphology etc.) and what are the limitations of these measurements with this new method.

**Fit for purpose** describes if the method used to answer the scientific question asked is relevant or meaningful as well as capable of answering within acceptable limitations. This term encompasses all the other criteria required for both regulatory and exploratory uses of this method; relevance, context of use and reliability/robustness.

## OoC in preclinical drug development

OoC systems can have human relevance and potentially a predictive capacity higher than animal models [6–9]. In addition, unlike *in vivo* models, they have the capacity to identify, record, and assess the effects of a singular parameter (force, flow, specific cell type, etc.) on the biological model by adding or subtracting it to/from the system in a functional way [10]. Because of this, there is significant interest in adapting these models to replace or complement *in vivo* models. This intention of course brings OoC models in direct proximity to regulatory oversight. Currently, there is no OoC model that has achieved full regulatory acceptance except for extremely limited case by case situations where OoC data are included as part of the application package. Given the speed of the exponentially growing innovation in OoC models, several platforms have been/are being developed and this plurality of technologies will make a consistent qualification process a significant challenge.

In situations where there are different testing approaches developed for the same CoU, regulators have, instead of recommending the use of one or more particular methods, proposed qualification and performance criteria to demonstrate fit for regulatory use (see ICH S5(R3)) [11]. In the context of OoC systems, as there are many different platforms in a particular field and considering continuous technological progress, regulators could follow the same approach. In fields with such great diversity, regulators prefer to define ‘*qualification criteria that are needed to accept the models'*. However, it is still not straightforward to draw a qualification criterion with meaningful points that is applicable to the whole field and every possible CoU which brings us to the topic of standardisation as a regulatory instrument [12]. Although it was outside of the scope of this workshop, the authors offer a potential solution to this dilemma by distinguishing between a basic qualification and a functional qualification which would change according to the CoU. Functional validation requires a qualification criterion to be established and trying to develop this for each individual CoU is both unrealistic and unnecessary. On the other hand, a qualification criterion for the basic functionality of each healthy tissue that is applicable to all OoC systems can be established. This is the case for a liver-on-chip where a definition of basic functionality criteria was limited to the amount of urea and albumin secretions [13]. From there, one can aim to qualify the same model for many different CoUs. For example, a liver-on-chip model of NASH will require a different set of complex functionality testing than an off-target cytotoxicity testing of CAR-Ts on the same liver-on-chip. Although the need or the extent of the standardisation wasn`t discussed at this workshop, the authors recognise the value of the current efforts in the field towards standardisation which started with drawing the European Organ on Chip roadmap in 2019 [14]. In 2021, the European Commission's Joint Research Centre (JRC) has taken the first step towards this aim by organising the Putting Science into Standards (PSIS) workshop together with the European Standardisation Organisations CEN and CENELEC (CEN-CNLC) [15]. In that workshop, initial priority areas were established such as an agreed uniform terminology for better communication and data sharing, standards around the devices themselves from materials (proteins, media, cells, chemicals etc.) to manufacturing which are a prerequisite before CoU qualification as well as standardising the evaluation of the biological performance, basic or functional qualification. In addition to the standardisation of the terminology and device classification, the authors agree there are aspects of manufacturing where establishing standards will ensure batch-to-batch reproducibility. Furthermore, having standards in place may ensure that both the chips and their accessories (e.g. pumps, tubing) fit with current lab equipment and increase their general uptake. However, applying standardisation across the whole field is far from straightforward. Lower Technology Readiness Level (TRL) systems have higher compatibility with existing accessory equipment as they utilise what is available off the shelf, but higher TRL systems rely more on customised accessory engineering aspects to improve their ease of use. For the latter, improving their compatibility with existing systems will be financially challenging. Hence the authors recommend a more realistic approach to increasing standardisation across the OoC field is a greater focus on increasing general uptake [16].

Ideally, for each CoU there should be an *accepted gold standard* as the reference for comparison for the OoC systems and a clear definition of *essential parameters/endpoints,* preferentially clinical biomarkers. The benefit of using clinical biomarkers are because these are established and validated biomarkers that are already in use. It is also not clear for each CoU what constitutes a gold standard and there is also a need for a discussion on what is the gold standard in what situation reliability. A consensus between regulators and the end-users/scientific community is required to define the gold standard. Identifying a gold standard for each CoU is not always possible especially with the developing industry pipeline focusing on human specific and novel modalities such as cell therapies where a consensus is lacking, a gold standard is not available or do not readily apply. Here, it becomes even more important to define what is being compared against. In such cases in conjunction with the need to define key aspects of the CoU, developing an *endpoint specific performance criterion with a list of commercially available, well known, positive and negative reference compounds* is key to the qualification of OoCs in general. That way the limitations of the model can be established and thereby the scope of validity or qualification for that CoU is reached. Establishing this standardised performance criterion for the CoU is not only a way to ensure the data are comparable between different platforms but also to layout where the confidence to the translatability of the model will be highest. Here, the identification of the most clinically relevant endpoints is critical to secure translatability from *in vitro* to the clinic as the whole purpose of the exercise is to establish a line of sight at bench to bedside (and vice versa, to enable a retrospective evaluation of the *in vitro* method against clinical data, once available). Additionally, given the rapid advances of the OoC field, the list of relevant endpoints to be extracted from OoCs need to be regularly updated to integrate new, promising tissue-level read-outs with high clinical relevance [10].

The *qualification* requires clarity on the scientific question(s) that the model is expected to answer, namely the CoU; once this condition is fulfilled, it is possible to clearly define where in the whole regulatory realm the model can be used and what are its limitations as described above. Together with the *scope of validity*, the *reliability* of the model and its *relevance* to the scientific question can be established. Of course, standardisation is particularly important to ensure reproducibility, a condition that is common to every model. Fulfilling these key aspects are the fundamental requirement for any novel method to reach regulatory acceptance.

In preclinical drug development, the main applications for OoC are species specific responses for safety pharmacology testing; absorption, distribution, metabolism, excretion, and toxicity (ADME-Tox); in identifying the right patient population for enhanced efficacy and in some potentially rare cases of developing personalised medicines such as adoptive cell therapies. Both ADME, toxicology/safety pharmacology assessment and personalised medicine are the fundamental areas where extensive qualification is needed as these data are not kept internal like the early drug discovery data but directly submitted to the regulatory agencies as part of the regulatory dossiers.

There is also considerable concern about building OoC based tissue models containing multiple different cells from different donors. This concern is especially valid when immune cells are included in the model, either tissue resident or circulating, and the scientific question asked is also immune response related where the donor mismatch may activate these cells leading to a response that is not relevant. This is of course a consequence of the challenge of developing fully autologous *in vitro* models based on primary cells, which are usually the preferred choice due to their physiological relevance. However, iPSCs differentiated into tissue derived mature cells can enable the development of fully autologous models. Unfortunately, maturation of certain iPSC derived cells is still not complete, and more progress is required in order to develop and increase the availability of fully autologous OoC models with multiple different cell types.

## Current gaps

As different end-users need to address either similar, albeit not identical or very different types of scientific questions, the same model requires tweaks on a case-by-case basis such that the data must be generated in-line with the respective end-users requests. This makes a harmonised model qualification process particularly time and resource consuming for both the technology developers and the end-users.

The fundamental reason for such a fragmented situation is the enormous innovation in the field that is shared between small technology developers and academics. The range and the scope of the innovation in the field is of course a benefit in the long term as it is benefiting from the full potential of scientific creativity but at the same time this diversity hampers the harmonisation of the protocols, or other key parameters between systems.

Another disadvantage of this fragmented innovation is that all models look quite promising at the beginning, and it is therefore very difficult to judge what constitutes a quick win for bringing a model to its next phase. Hence as end-users, pharmaceutical companies need to simultaneously monitor many different technologies, testing them for different applications and proof-of-concept studies to generate *ad hoc* data. This is very resource intensive for both the end-users and the technology developers when different end-users request similar qualification experiments with a different set of reference compounds or endpoints/biomarkers, which is very costly for the industry and also puts a significant strain on the limited resources from the technology developers. This situation further highlights the importance of establishing standardised lists of reference compounds for a series of the most common CoU scenarios so that technology developers can create this data once and can allocate their resources to refining available models or to developing new ones.

A similar hurdle emerges in the current need for developing separate models for assessing safety and efficacy. This scenario is more often experienced by the end-users and doubles the amount of effort to create, evaluate and qualify two separate models. It is more efficient to combine efficacy and safety models and the result is also more relevant to real world data. For example, a bone marrow and tumour-on-chip combination will reveal whether the effective dose for a tumour has a safety implication on the bone marrow. A combined approach potentially also be embraced by the regulators, given that it provides more holistic approach usually only found in animal models.

Another important challenge emerging from this fragmentation in the field is communication and interaction between different stakeholders. The need to communicate and exchange experience and expertise have led to different networks, consortia, and hubs in a multitude of geographical and professional locations. The lack of crosstalk between networks, however, is a hurdle preventing replication and harmonisation of protocols and processes. Recently, this challenge has only increased as the COVID-19 pandemic has limited the opportunities to interact.

## Future directions

Although research in OoC technology is still too siloed, there are signs of improvement. For example, the Microphysiological Systems working group within the US Innovation and Quality (IQ) Consortium brings end-users together, currently with 21 companies involved including pharmaceutical and biotechnology organisations [17]. The need for a consortium to bring together technology developers was also highlighted and soon after this conference, The North American 3Rs Collaborative (NA3RsC) MPS Initiative has taken over that role with currently 55 representatives from 28 commercially available MPS developers [18]. With the main aim of increasing the adoption of MPS technologies by stakeholders, NA3RsC founded a dedicated an initiative to facilitate regulatory acceptance as part of that overarching goal. On the academic side, the European Organ-on-Chip Society (EUROoCS) has been a key organisation connecting academic innovators under the light of regulatory and industry input with dedicated advisory boards in place for both [19]. A similar network in U.K., Organ-On-a-Chip Technologies Network, is aimed to nurture and expand the OoC research by facilitating academic and industry interaction [20]. Many other similar national OoC networks are developing over the last few years. Additionally, The NC3Rs open innovation programme CRACK-IT provides a unique mechanism to support cross sector collaboration in OoC technology development and application [21].

As of now, at least on the EU-US axis, three legs of the table are in place but a consortium or a closer interaction between the regulators are still missing globally. A consortium of model developers, regulators and end-users would enable creating a list of reference compounds for specific CoUs. Here input especially from regulators could be very useful as they have oversight on every drug modality and can assist with giving insight into the tools needed or in identifying gaps. Also, discussions could be held to decide the type and amount of data needed for qualification in relation to the specific CoU. For example, qualification requirements might differ if data generated with an OoC model support mechanistic underpinning or whether they are providing pivotal — guideline driven data. A possible next stage would be testing centres independently running qualification against reference compounds which would increase the confidence and facilitate the route to regulatory acceptance. Independent testing centres have been utilised in the US as Tissue Chip Testing Centers; these were an early attempt to independently evaluate different platforms. The experience highlighted the numerous practical issues with concept, which include a lack of completely standardised methods and reference compounds, commercial considerations limiting the ability to compare the performance of each OoC model against each other.

What is most important in this process is to involve the regulators in the discussion as early as possible and especially during the model qualification process. Early dialogue and sharing these data enable the regulators to familiarise themselves with new models from the start of their development and increases their understanding of the data generated and their confidence in the model used. In addition, regulators can provide insight in possible regulatory relevant CoU and definition of qualification needs (criteria, reference compounds, etc). Additional methods to increase the familiarity of the regulators with these models include: (1) collaborative scientific research between regulators, developers and pharmaceutical companies, (2) open training by technology developers in a pre-competitive entirely educational manner. The important aspect here is to keep the training continuous and consistent, for example by using the same training materials.

**Figure 1.**
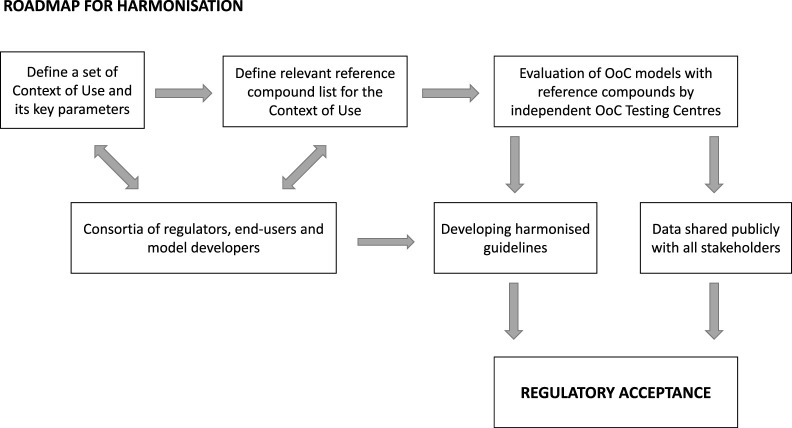
Roadmap towards achieving harmonisation and regulatory acceptance for OoC models.

Although early interaction with the regulators is going to increase their familiarity with the technology as it develops, end-users may be hesitant to follow this route unless a clear process is put in place to ensure the generated data are not automatically considered for product-related regulatory decision making purposes since drug developers are generally reluctant to generate such data in unvalidated models. To avoid this happening, a so-called *safe harbour* approach has been put in place by the EMA to encourage the voluntary submission of data generated with new models. Data submission is fostered through the *EMA Innovation Task Force* and novel 3Rs testing approach may be submitted to the EMA in accordance with the procedure described in the guideline on *Qualification of Novel Methodologies for Drug Development* and is assessed by the *EMA Scientific Advice Working Party.* The FDA also strongly encourages data submission through many different routes such as Innovative Science and Technology Approaches for New Drugs (ISTAND) Pilot Program from the Center for Drug Evaluation and Research's (CDER) or the FDA webinar series on alternative methods and others. Although there have already been some OoC derived data submissions to regulators, the path forward requires a wider adoption which is outlined in Figure 1.

The first step towards harmonisation will be to define the CoUs to focus on, which already represents a big challenge. Here both regulators and end-users together should indicate where the main gaps are. The next step will be for regulators and end-users again to define together a set of appropriate, data rich and commercially available reference compounds for each CoU. A significant amount of funding would be required to establish independent OoC Testing Centres that could qualify different platforms according to reference compounds and performance of these models in relation to the endpoints, thereby establishing the *qualification* for each platform. Substantial work would be required to ensure the testing centres are trusted by all stakeholders and are viewed as entirely independent. It is essential that the data are shared amongst all stakeholders to increase confidence and to facilitate the development of harmonised guidelines for the qualification of forthcoming new models in the field. It is hoped that the entire process will lead to a unified global regulatory acceptance.

## Conclusion

Despite the multiple topics discussed, the main take-home messages of the conference can be summarised in that the OoC field has reached its peak of expectations and key challenges are to be addressed to avoid potential disillusionment and to be able to reach to the next phase of increased general uptake by all relevant industries.

Fragmentation of the field results from strong innovation leading to resource intensive qualification both by the technology developers and end-users alike. Overall a lack of standardisation in the field is leading to a multi-headed approach which should be consolidated into a more focused effort. The field has reached a critical phase and the decisions made for the short-term will affect the longer-term success of this technology. However, there has been a strong common ground identified by the meeting attendees: model developers, end-users, and regulators are all working towards making the pre-clinical models more human relevant and increase their predictive capacity. The scientific robustness is at the heart of everyone's interest not least the regulatory agencies, who are keen to embrace the concept of ‘*qualification in relation to context of use'*. If all key stakeholders work together in the same direction, the goal of wider adoption of OoC models by various industries (pharmaceutical, chemical, cosmetics, food safety etc.) and the regulatory agencies in replacement of animal models can be reached.

## Perspectives

- Organ on chip (OoC) technologies are progressively achieving a level of complexity that had previously been limited to *in vivo* models.
- There is a clear drive, led by the FDA, EMA, EU National Competent Authorities and others, to accelerate the development, use and qualification of non-animal models, where OoC is a suitable alternative.
- Strong innovation is leading to fragmentation in the field which is manifesting itself as discrete and uncoordinated efforts in the qualification of these models.

## Abbreviations

CIVMs: Complex In Vitro Models
NA3RsC: North American 3Rs Collaborative
TRL: Technology Readiness Level
